# Brain pH Measurement Using AACID CEST MRI Incorporating the 2 ppm Amine Resonance

**DOI:** 10.3390/tomography8020060

**Published:** 2022-03-09

**Authors:** Mohammed Albatany, Susan Meakin, Robert Bartha

**Affiliations:** 1 Centre of Functional and Metabolic Mapping, Robarts Research Institute, The University of Western Ontario, London, ON N6A 3K7, Canada; malbatan@uwo.ca; 2 Department of Biochemistry, Schulich School of Medicine and Dentistry, The University of Western Ontario, London, ON N6A 3K7, Canada; smeakin@uwo.ca; 3 Department of Medical Biophysics, Schulich School of Medicine and Dentistry, The University of Western Ontario, London, ON N6A 3K7, Canada

**Keywords:** Brain pH, AACID, CEST MRI, 2 ppm amine resonance, cancer, glioblastoma multiforme

## Abstract

Many pathological conditions lead to altered intracellular pH (pH_i_) disrupting normal cellular functions. The chemical exchange saturation transfer (CEST) method, known as Amine and Amide Concentration Independent Detection (AACID), can produce image contrast that is predominantly dependent on tissue intracellular pH_i_. The AACID value is linearly related to the ratio of the 3.5 ppm amide CEST effect and the 2.75 ppm amine CEST effect in the physiological range. However, the amine CEST effect at 2 ppm is often more clearly defined in vivo, and may provide greater sensitivity to pH changes. The purpose of the current study was to compare AACID measurement precision utilizing the 2.0 and 2.75 ppm amine CEST effects. We hypothesized that the 2.0 ppm amine CEST resonance would produce measurements with greater sensitivity to pH *changes*. In the current study, we compare the range of the AACID values obtained in 24 mice with brain tumors and in normal tissue using the 2 ppm and 2.75 ppm amine resonances. All CEST data were acquired on a 9.4T MRI scanner. The AACID measurement range increased by 39% when using the 2 ppm amine resonance compared to the 2.75 ppm resonance, with decreased measurement variability across the brain. These data indicate that in vivo pH measurements made using AACID CEST can be enhanced by incorporating the 2 ppm amine resonance. This approach should be considered for pH measurements made over short intervals when no changes are expected in the concentration of metabolites that contribute to the 2 ppm amine resonance.

## 1. Introduction

Intracellular pH plays an important role in many physiological processes, including apoptosis, cell proliferation, and protein interactions, and it is altered in several disease states. In cancer, altered intracellular and extracellular pH gradients can lead to drug resistance [1,2]. Chemical exchange saturation transfer (CEST) can produce image contrast that is dependent on tissue pH [3,4,5,6], and can be used to non-invasively study cellular pH under various conditions. More specifically, amide proton transfer (APT) efficiency varies with pH, providing sensitivity to this physiological parameter. Although the measurement of the APT CEST effect depends on several factors including the amide proton concentration, water concentration, and the relaxivity (R_1_) of bulk water [6], this contrast has been successfully used to identify ischemic tissue following acute stroke [7,8,9,10,11,12] and to study cancer [13,14,15,16,17].

In previous work [18] using 9.4T MRI, we have demonstrated that the ratio of the 3.5 ppm amide CEST effect to the 2.75 ppm amine CEST effect varies linearly with pH in the physiological range, and is largely independent of protein concentration and temperature [18]. This ratiometric CEST method called Amine and Amide Concentration Independent Detection (AACID) [18] is sensitive to the acidification of the brain following stroke, and to the increased intracellular pH in brain tumors [19,20]. The method has also been used to measure the magnitude of acute tumor acidification using the pharmacologic agents lonidamine, topiramate, dichloroacetate, cariporide, and quercetin [19,20,21,22,23].

However, due to the fast exchange rate of amine protons in vivo, the 2.75 amine CEST effect provides only a small response to changes in pH when using the low amplitude, long duration saturation schemes typically used for amide proton detection. In contrast, the 2.0 ppm amine peak is better defined and produces a greater in vivo pH response [24,25]. The CEST effect at 2.0 ppm is largely associated with creatine [24,25] and guanidinium [26], and has been shown to correlate with the creatine concentration measured by ^1^H-MRS [25]. A recent study demonstrated that reduced creatine CEST measured at 2 ppm could help differentiate aggressive from non-aggressive gliomas [26]. Given that the 2.0 ppm amine peak is more easily detected in vivo, the use of this peak could increase the sensitivity of short-interval in vivo AACID ratiometric measurements compared to the use of the 2.75 ppm amine peak, particularly for the rapid measurement of pH changes after administration of pharmacologic agents, where short term changes in creatine and guanidinium are not expected.

The purpose of the current study was to investigate whether AACID CEST measurements incorporating the 2 ppm amine resonance would have greater sensitivity compared to measurements made using the 2.75 ppm resonance in brain tumors and contralateral tissue. Protamine phantoms were first used to determine whether there was a linear relationship between the AACID value measured using the 2.0 ppm amine resonance and pH within the physiological pH range, followed by a re-examination of AACID CEST data previously acquired in mice with brain tumors.

## 2. Experimental

### 2.1. Phantom Preparation

To validate the use of the 2 ppm amine resonance in the calculation of the AACID ratio, we examined whether the AACID response was linear over the physiological pH range. A series of protamine (EMD Millipore, Oakville, ON, Canada) phantoms were created in 5 mm diameter tubes with pH values ranging from 6–8: 6.12, 6.32, 6.56, 6.78, 7.12, 7.44, 7.71, and 8.03. Protamine produces a 2 ppm amine CEST peak analogous to that observed in the human brain [27,28]. The phantoms contained protamine at a concentration of 12 mg/mL dissolved in phosphate buffered saline. All phantoms were scanned simultaneously at 37 °C.

To characterize the magnitude of the CEST effect at 2 ppm as a function of creatine concentration at constant pH, a series of creatine (Sigma Aldrich, Oakville, ON, Canada) and bovine serum albumin (BSA, Sigma-Aldrich, St. Louis, MO, USA) phantoms were used. These phantoms were created in 5 mm diameter tubes with creatine concentrations ranging from 0–20 mM, mixed in 10% bovine serum albumin (BSA) at pH 7. Creatine contributes to the 2 ppm amine CEST peak observed in vivo [24,25]. All phantoms were scanned simultaneously at 37 °C.

### 2.2. Animal Tumor Preparation

All data utilized in this work were obtained from previous studies where animal procedures were approved by the Animal Care Committee at Western University. The animal GBM model used in these studies was previously described [19,21,22,23] but is provided here for completeness. GBM brain tumors were induced in all twenty-four (N = 24), Crl:Nu-Foxn1^Nu^ (NU/NU) mice (22–27 g, Charles River Laboratories, Saint-Constant, QC, Canada) included in this study. Briefly, U87MG glioma cells established from a human GBM (ATCC; Rockville, MD, USA) [29] were grown at 37 °C using Dulbecco’s modified Eagles’ medium supplemented with 10% fetal bovine serum (Wisent Inc., St-Jean-Baptiste, QC, Canada). Cells were grown in a humidified incubator with 5% CO_2_ and passaged twice a week. The U87MG cells were washed and dissociated with versene solution (PBS plus 0.5 mM EDTA). The cells were then washed twice with PBS, counted, and re-suspended to produce a final concentration of 1 × 10^5^ cells in 2 mL PBS prior to injection. The mice were anesthetized using 4% inhaled isoflurane and then maintained under anesthesia using 1.5% isoflurane. A stereotactic head frame (Stoelting instruments, Wood Dale, IL, USA) was used to guide cell injection. After exposing the bregma, a 1 mm diameter hole was drilled 1 mm anterior and 2 mm lateral to the bregma. A Hamilton syringe (Reno, NV, USA) with a 27-gauge needle attached was used to inject 2 µL of U87MG cells 3 mm deep from the bregma at a rate of 0.5 μL/min, into the right frontal lobe [19,21,22,23].

### 2.3. Preparation of Mice for In Vivo Imaging

CEST data from all mice included in this study have been previously used to produce AACID maps using the 2.75 ppm resonance [19,21,22,23]. In the current study, we re-examined the data from these mice to compare the AACID CEST measurements using the 2.0 ppm and 2.75 ppm amine resonances. The imaging protocol that was used is summarized as follows. The mouse head was placed within a 30 mm millipede volume coil in a dedicated small animal MRI scanner (9.4T, Agilent, Palo Alto, CA, USA) approximately fifteen (15 ± 1) days after the injection of cancer cell. Mice were anesthetized initially using 4% inhaled isoflurane in oxygen and then maintained using 1.5%–2.5% isoflurane in oxygen. A custom-built MRI-compatible stage was used to secure each mouse, and a bite bar was used to further reduce motion in the head [20]. Respiratory motion was also reduced using surgical tape. A rectal temperature probe was used to monitor temperature, and a respiratory sensor pad connected to a pressure transducer placed on the thoracic region was used to monitor breathing. Warm air blown over the animal using a model 1025 small-animal monitoring and gating system (SA Instruments Inc., Stony Brook, NY, USA) maintained body temperature between 36.9–37.1 °C during imaging. Animals were sacrificed immediately after MR imaging. Data from a single (NU/NU) mouse (Charles River Laboratories, Saint-Constant, QC, Canada) without tumor was also included for comparison.

### 2.4. Magnetic Resonance Imaging

All phantoms (protamine and creatine phantoms) were scanned using the same 30 mm millipede volume coil used to scan the mice. This study reanalyzed the in vivo data from previously published studies that used the following imaging protocol [19,21,22]. T_2_-weighted images were acquired to visualize the phantoms or the tumor in mice using a fast spin-echo pulse sequence (FSE) with the following parameters: slice thickness = 1 mm, matrix size = 128 × 128, FOV = 25.6 × 25.6 mm^2^, ETL = 4, effective TE = 40 ms, and TR/TE = 3000/10 ms. These T_2_-weighted images were used to position the CEST slab (4 mm thickness) for maximum phantom or tumor coverage. An FSE pulse sequence preceded by a continuous wave radiofrequency (RF) pulse (1.5 µT amplitude and 4 s duration) was used to acquire CEST images (slice thickness = 4 mm, matrix size = 64 × 64, FOV = 25.6 × 25.6 mm^2^, ETL = 32, effective TE = 7 ms, TR = 7000). A series of 49 CEST images was acquired at sampling saturation frequencies from 1.2 to 4.5 (∆ = 0.1) ppm, from 5.4 to 6.6 (∆ = 0.1) ppm, and at −1000 and 1000 ppm as references. Two series of CEST images were acquired to increase the signal-to-noise ratio. The water saturation shift referencing (WASSR) technique was used for correction of the B_0_ shifts [30]. A series of 37 WASSR CEST images were acquired with saturation frequencies linearly spaced between −0.6−0.6 ppm. The same pulse sequence as the AACID CEST acquisition was used for WASSR, except it was preceded by a low amplitude (0.2 µT) short duration (100 ms) RF saturation pulse.

### 2.5. CEST Data Processing

Custom MATLAB (Mathworks, Natick, MA, USA) code was used to analyze all CEST data on a pixel-by-pixel basis as previously described [19,21,22,23]. The WASSR and CEST spectra associated with each pixel were interpolated to 1-Hz resolution. The “smooth” algorithm in the MATLAB curve fitting toolbox was used to smooth all the CEST spectra. To correct the shifts induced by B_0_ field variations within the sample, the CEST spectrum was frequency shifted for each pixel so that the water signal was centered at 0 ppm, using the corresponding WASSR spectrum. This correction ensured that B_0_ variations were corrected prior to summing the spectra from pixels within defined regions of interest. After the B_0_ corrections, the CEST spectra from both acquisitions were added to increase the signal-to-noise ratio. No B_1_ correction was applied [19] because we have previously shown that the B_1_ variation in the CEST slice was not appreciable [19].

#### 2.5.1. Calculation of AACID Values

The ratio of the CEST effects at 2.75 ppm (amine protons) and at 3.50 ppm (amide protons), normalized by the CEST effect at 6.0 ppm, gives the AACID_2.75_ value, as shown in Equation (1) [18].

(1) $${\mathrm{AACID}}_{2.75}=\frac{M_{Z3.5\;\mathrm{ppm}}\times \left(M_{Z6.0\;\mathrm{ppm}}-M_{Z2.75\;\mathrm{ppm}}\right)}{M_{Z2.75\;\mathrm{ppm}}\times \left(M_{Z6.0\;\mathrm{ppm}}-M_{Z3.5\;\mathrm{ppm}}\right)}$$

The AACID_2.0_ value (Equation (2)) was calculated by substituting the CEST effect at 2.0 ppm for the CEST effect at 2.75 ppm in Equation (1).

(2) $${\mathrm{AACID}}_{2.0}=\frac{M_{Z3.5\;\mathrm{ppm}}\times \left(M_{Z6.0\;\mathrm{ppm}}-M_{Z2.0\;\mathrm{ppm}}\right)}{M_{Z2.0\;\mathrm{ppm}}\times \left(M_{Z6.0\;\mathrm{ppm}}-M_{Z3.5\;\mathrm{ppm}}\right)}$$

The mouse brain AACID_2.0_ maps were normalized to the average AACID_2.0_ value measured in the contralateral ROIs of all 24 mice. The same approach was used to normalize the mouse brain AACID_2.75_ maps using the average AACID_2.75_ value. This approach simplified the visual assessment of the quantitative AACID maps.

#### 2.5.2. Statistical Analysis

Contralateral tissue and tumor tissue regions of interest (ROIs) were manually drawn in each mouse brain by M.A., guided by the T_2_-weighted images. The ROIs were drawn using the MATLAB “roipoly” function and the average AACID values within each ROI were calculated. A paired Student’s *t*-test was used to identify differences in average AACID values between the U87MG tumors and the contralateral tissue in the 24 mice when using the 2.75 ppm and 2.0 ppm amine resonances (GraphPad Prism Version 9.3.1.471 for Windows, GraphPad Software, San Diego, CA, USA). In all comparisons, *p* < 0.05 was considered statistically significant.

## 3. Results

**Figure 1:**
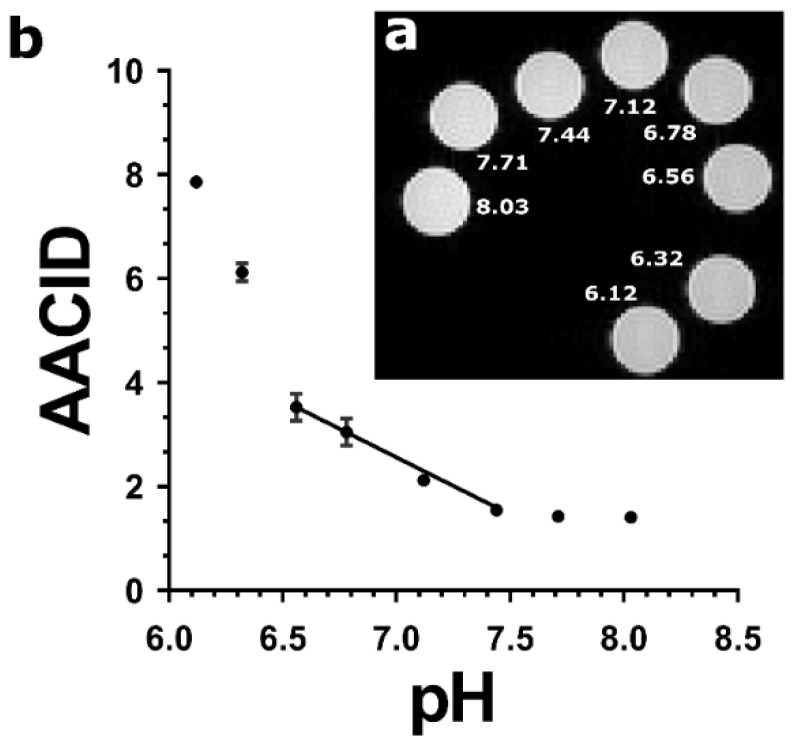
(**a**) T_2_-weighted FSE image showing cross sections of eight protamine samples at pH values ranging from 6.12 to 8.03 in NMR tubes scanned at 9.4T and at 37 °C. (**b**) AACID values calculated using the 2 ppm amine resonance as a function of pH in protamine samples. The relationship between AACID and pH can be approximated as linear between pH 6.6–7.4. The AACID value is most sensitive to change at low pH (6.1–6.6) and does not change appreciably above pH 7.4. Error bars represent the standard error of the mean within each NMR tube.

A sample image of the arrangement of the protamine phantoms in the MRI scanner is provided in (Figure 1a). In the protamine solutions, the AACID_2.0_ value calculated using Equation (2) varied nonlinearly as a function of pH (Figure 1b) over the large range of pH values tested, showing greater pH sensitivity at a low pH (below 6.6) and little sensitivity above pH 7.4. The pH response in the range from pH 6.6–7.4 could be approximated as a linear function (indicated by the superimposed line).

**Figure 2:**
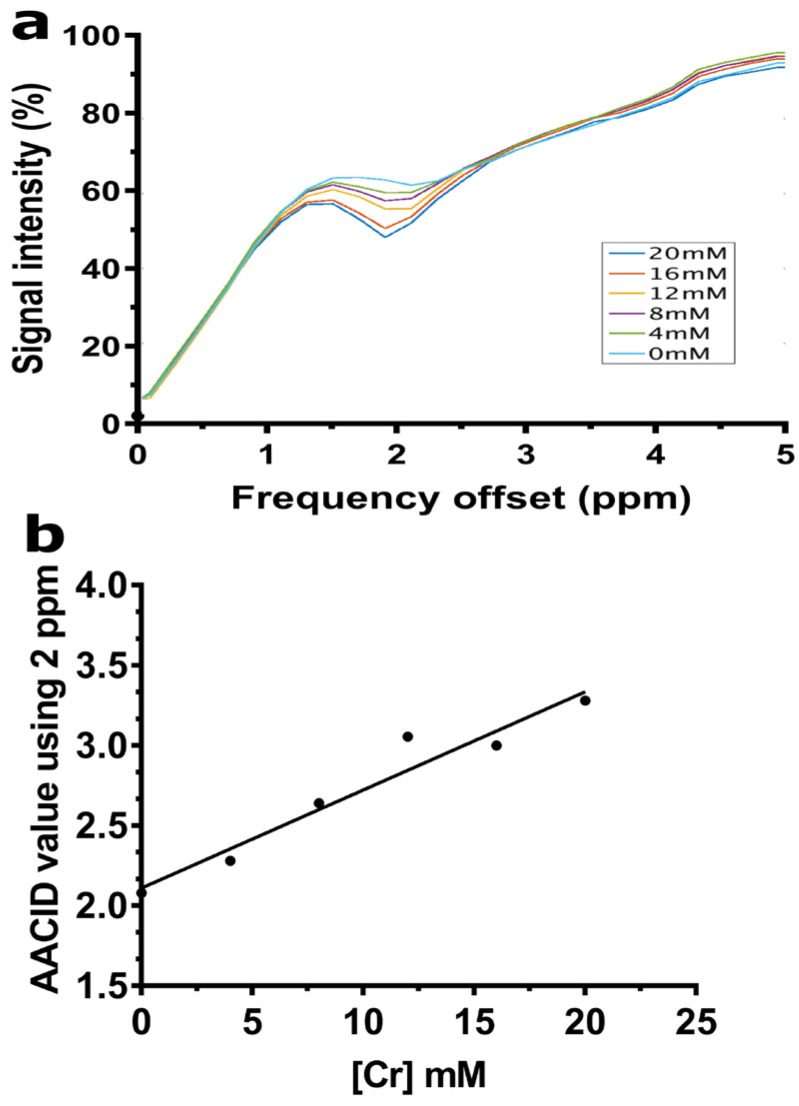
(**a**) CEST spectra acquired at 9.4T are shown for samples with Cr concentrations ranging from 0–20 mM mixed in 10% bovine serum albumin (BSA) at 37 °C and pH 7. (**b**) The AACID value shows a linear increase proportional to the increase in creatine concentration when using the 2 ppm resonance at constant pH.

The creatine CEST effect was concentration-dependent and easily detected when using the 1.5 µT and 4 s duration saturation pulse used in the AACID acquisition scheme. The CEST peak observed at 2 ppm showed a greater CEST effect as the creatine concentration increased (Figure 2a). Consequently, the AACID values increase linearly as expected as the creatine concentration increased (Figure 2b).

**Figure 3:**
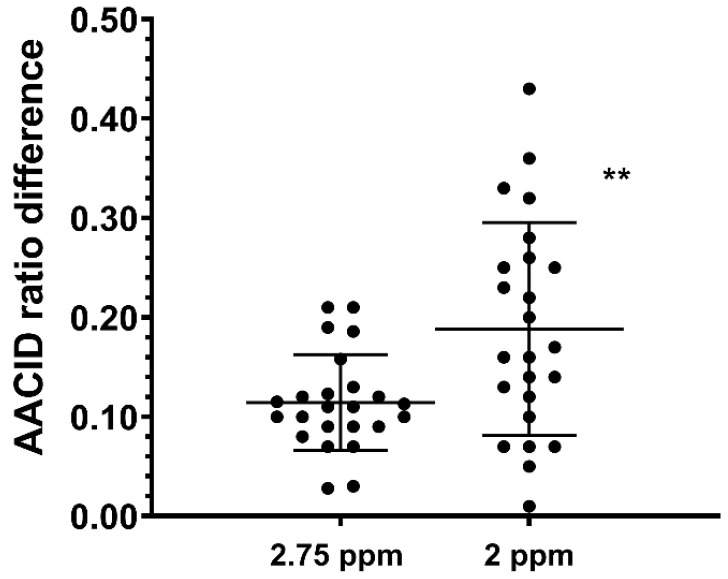
Average difference in AACID values between U87MG tumors and contralateral tissue in 24 mice when using the 2.75 ppm and 2.0 ppm amine resonances. ** indicates *p* < 0.001 in a repeated measures two-tailed t-test. Error bars represent the standard error of the mean.

The average AACID_2.75_ value in U87MG tumors was 1.15 ± 0.05, while the average AACID_2.75_ value in contralateral tissue was 1.27 ± 0.041. The average AACID_2.0_ value in U87MG tumors was 1.93 ± 0.11 while the average AACID_2.0_ value in contralateral tissue was 2.12 ± 0.10. In both cases, the AACID value in the tumor was significantly lower (*p* < 0.05) than the contralateral side, indicating a more basic pH in the tumor. The average difference in AACID values between U87MG tumors and contralateral tissue in the 24 mice studied was 39% greater when using the 2 ppm amine resonance compared to the 2.75 ppm resonance (Figure 3). Therefore, use of the 2 ppm amine resonance provides a greater range for the AACID measurement.

**Figure 4:**
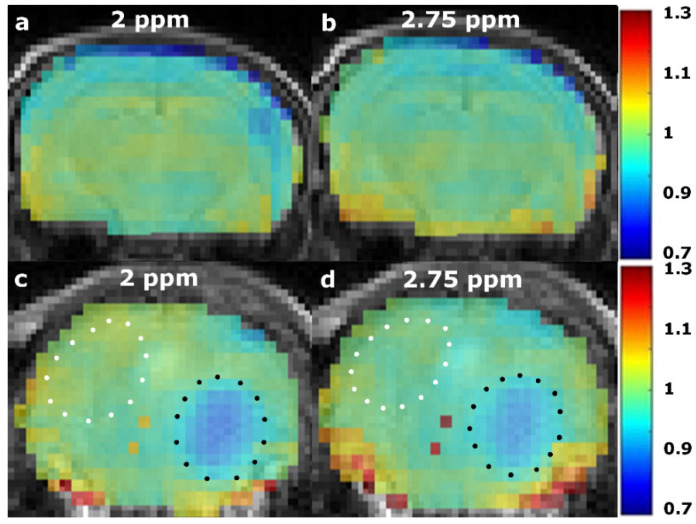
Normalized AACID maps obtained in a single healthy NU/NU mouse brain using the 2 ppm amine resonance (**a**), compared to the 2.75 ppm (**b**) amine resonance. Normalized AACID map of a NU/NU mouse with a brain tumor using the 2 ppm amine resonance (**c**), compared to the 2.75 ppm (**d**) amine resonance. Typical manually defined ROIs are shown on the contralateral side (white dotted line), and over the tumor (black dotted line).

Example AACID CEST maps from a single mouse are provided in Figure 4 when using the 2 ppm amine resonance (Figure 4a) and the 2.75 ppm amine resonance (Figure 4b). These normalized AACID maps appear more symmetric when using the 2 ppm amine resonance (Figure 4a) compared to the 2.75 ppm amine resonance (Figure 4b). Similarly, AACID CEST maps from a single mouse with a brain tumor are shown when using the 2 ppm amine resonance (Figure 4c) and the 2.75 ppm amine resonance (Figure 4d). The dark circular region of low AACID value (elevated pH_i_) represents the tumor region. The tumor was more easily identified in the AACID maps when using the 2 ppm amine resonance (Figure 4c) compared to the 2.75 ppm amine resonance (Figure 4d).

## 4. Discussion

The objective of this work was to determine whether the use of the 2 ppm amine resonance could increase the sensitivity of the AACID ratiometric measurement of intracellular pH. In protamine phantoms, it was shown that the AACID_2.0_ value obtained using the 2 ppm resonance could be approximated by a linear function between pH 6.6 and 7.4 at 37 °C. Comparing the in vivo AACID_2.0_ measurements in tumor and in contralateral tissue to the AACID_2.75_ measurements, the 2 ppm amine resonance increased the AACID range by 39% compared to the 2.75 ppm resonance. This greater range suggests that using the 2 ppm amine resonance could improve AACID-based pH measurement in vivo. The main drawback of using the 2 ppm amine resonance is that it includes contributions from metabolites such as creatine (guanidinium protons), which can fluctuate in concentration in different tissue types. Therefore, the 2 ppm amine resonance should only be used in conditions where creatine concentration can be approximated as stable throughout the measurement.

Protamine is a small, arginine-rich protein that was used in the current study to model CEST contrast because it contains both exchangeable amine and amide protons that can be used in the AACID measurement. The protamine CEST spectra showed the expected peaks at 2.0 ppm from amine protons and at 3.5 ppm from amide protons. The approximately linear relationship observed between the AACID_2.0_ value and pH in the protamine phantoms within the physiological pH range (6.6–7.4) is not required, but ensures that changes in the AACID value can be easily related to changes in pH. Such a linear response was previously shown for the AACID value obtained when using the 2.75 ppm amine resonance in bovine serum albumin [18]. The previously observed pH_i_ dependent contrast was also insensitive to macromolecule concentration, tissue temperature, and bulk water T_1_ relaxation [18]. In vivo, the 2 ppm resonance is mostly produced by creatine, although there are also contributions from other metabolites that have chemical shifts near 2 ppm, such as phosphocreatine, adenosine triphosphate, guanidinium and adenosine diphosphate. However, these metabolites have a slower amine proton exchange rate at physiological pH compared to creatine [31]. Therefore, the in vivo 2 ppm CEST signal can be mostly attributed to creatine [26,31,32].

To estimate the absolute pH change indicated by a change to the AACID_2.0_ measurement, the AACID_2.0_ values can be calibrated using the literature values of pH in tumors and healthy tissue. For example, the average AACID_2.0_ measurement in tumors from all 24 mice can be equated to 7.3, the average pH previously measured in brain tumors [33,34,35,36,37,38]. Similarly, the average AACID_2.0_ measurement on the contralateral side can be equated to 7.0, the average pH previously found in normal brain tissue [33,34,35,36,37,38]. Since AACID_2.0_ is linearly-dependent on pH_i_ over this narrow range (Figure 1b), this approach to calibration can be used to estimate the absolute pH change in healthy tissue or a tumor following an intervention. However, as described above, the 2 ppm CEST signal is also dependent on creatine concentration [26,31]. Creatine concentration is known to be different in cancerous tissue and to change with tumor progression. Specifically, glioma had significantly lower Cr CEST, a feature that could help to differentiate gliomas with different aggressiveness [26]. Moreover, creatine concentration depends on the tissue cell type and cellular composition. Therefore, to avoid potential errors caused by differences in the tissue Cr concentration, the use of AACID-based pH measurement in vivo with the 2 ppm amine resonance is best suited only to measure the *change* in pH after drug treatments. The 2 ppm resonance should not be used to compare tissues or in conditions that could potentially involve changes in Cr concentration associated with disease progression [25,26].

Several limitations must be considered in this study. First, the scope of testing was limited to only one tumor model (U87 glioma). It is expected that different tumor types will produce different changes in the AACID measurement, potentially due to a different concentration of creatine within the tumors. Future studies that incorporate regional ^1^H magnetic resonance spectroscopy measurements of creatine within the tumors could be used to assess the dependency of the 2 ppm AACID CEST measurement on creatine concentrations. Similarly, our in vitro work was limited to protamine. Other protein solutions containing a different ratio of amine to amide group concentrations would be expected to show a different response of the AACID value to pH.

## 5. Conclusions

AACID measurements sensitive to tissue intracellular pH can be made with the 2.75 ppm or 2.0 ppm amine resonance. Use of the 2 ppm amine resonance can provide a greater measurement range compared to the 2.75 ppm amine resonance. However, this method includes contributions from metabolites such as creatine that can fluctuate in concentration in different tissue types. Therefore, use of the 2 ppm amine resonance can be advantageous, but it should be considered only when measuring pH changes under conditions where the metabolites that contribute to the 2 ppm amine signal are stable for the duration of the measurement.

## Data Availability

The data presented in this study are available on request from the corresponding author. The data are not publicly available due to ethical considerations.

## Abbreviations

GBM	glioblastoma multiforme
pH_i_	intracellular pH
Cr	creatine
CEST	chemical exchange saturation transfer
RF	radiofrequency
MTR_asym_	asymmetric magnetization transfer ratio
MT	magnetization transfer
AACID	amine and amide concentration-independent detection
FSE	fast spin-echo
WASSR	water saturation shift referencing
AFI	actual flip-angle imaging
ROI	region of interest
